# Performance Assessment of Portable SLAM-Based Systems for 3D Documentation of Historic Built Heritage

**DOI:** 10.3390/s26020657

**Published:** 2026-01-18

**Authors:** Valentina Bonora, Martina Colapietro

**Affiliations:** Department of Civil and Environmental Engineering, University of Florence, Via di Santa Marta 3, 50138 Firenze, Italy; valentina.bonora@unifi.it

**Keywords:** SLAM, built heritage, 3D models, preliminary seismic risk assessment

## Abstract

The rapid and reliable geometric documentation of historic built heritage is a key requirement for a wide range of conservation, analysis, and risk assessment activities. In recent years, portable and wearable Simultaneous Localization and Mapping (SLAM)-based systems have emerged as efficient tools for fast 3D data acquisition, offering significant advantages in terms of operational speed, accessibility, and flexibility. This paper presents an experimental performance assessment of three portable SLAM-based mobile mapping systems applied to the 3D documentation of historic religious buildings. Two historic parish churches in the Lunigiana region (Italy) are used as case studies to evaluate the systems under real-world conditions. The analysis focuses on key performance indicators relevant to metric documentation, including georeferencing accuracy, 3D model accuracy, point cloud density and resolution, and model completeness. The results highlight the capabilities and limitations of the tested systems, showing that all instruments can efficiently capture the primary geometries of complex historic buildings, while differences emerge in terms of accuracy, data consistency, and readability of architectural details. Although the work is framed within a broader research project addressing seismic vulnerability of historic structures, this contribution specifically focuses on the experimental evaluation of SLAM-based surveying performance. The results demonstrate that portable SLAM systems provide reliable geometric datasets suitable for preliminary documentation tasks and for supporting further multidisciplinary analyses, representing a valuable resource for the rapid 3D documentation of historic built heritage.

## 1. Introduction

Historic masonry religious buildings represent a significant portion of the built cultural heritage in seismic-prone regions and are widely recognized as highly vulnerable to earthquake-induced damage. Their response to earthquake actions is strongly influenced by construction techniques, material heterogeneity, geometric complexity, material discontinuities, temporal stratification, and geometric irregularities. As a result, damage is often characterized by the activation of local collapse mechanisms involving macro-elements such as façades, apses, bell towers, and vaulting systems. The identification of these mechanisms is a key step in simplified seismic vulnerability assessment methodologies commonly adopted for historic masonry structures and requires reliable geometric and morphological information, traditionally obtained through visual inspections and drawings and documents.

Expeditious vulnerability assessment procedures typically rely on visual inspections and in situ surveys to collect geometric, typological, and constructive information necessary to identify potential failure modes. While direct observation remains essential, it is often constrained by practical limitations such as accessibility, safety conditions, survey time, and the need for expert operators.

Recent advances in 3D surveying technologies have significantly transformed the documentation of built heritage. Among these, Simultaneous Localization and Mapping (SLAM)-based systems have emerged as a rapid and flexible solution for metric data acquisition, as they enable real-time generation of 3D point models, drastically reducing acquisition time while maintaining adequate geometric consistency. The availability of such 3D models offers new opportunities for integrating geometric documentation and structural assessment workflows. In particular, they enable the extraction of metric and spatial information relevant to seismic vulnerability analyses, including dimensions of structural elements, geometric proportions, articulation of macro-elements, and spatial relationships between load-bearing components.

This raises the question of whether SLAM-based 3D models can effectively support, or partially replace, traditional in situ observation in expeditious seismic vulnerability assessment procedures.

This paper investigates the reliability and applicability of SLAM-derived 3D models for the rapid seismic vulnerability assessment of historic masonry religious buildings. The study focuses on evaluating the level of detail and metric accuracy required to extract the information typically employed in simplified assessment methods. Beyond rapid assessment purposes, the paper highlights the role of SLAM-based 3D documentation as a foundational dataset for more advanced analyses, as numerical simulations and structural analyses, supporting a multi-scale and multidisciplinary approach to seismic risk assessment. In this framework, fast 3D surveying techniques act as a bridge between geometric documentation and structural interpretation, enhancing both efficiency and analytical potential.

## 2. State of the Art and Literature Review

In recent years, the three-dimensional documentation of built heritage has undergone a profound transformation, driven by the rapid development of technologies such as Digital Photogrammetry (DP) [1] and Terrestrial Laser Scanning (TLS) [2]. The integration of these two methods has proven highly effective due to their complementary characteristics.

More recently, a significant innovation in this field has been the introduction of Mobile Mapping Systems (MMSs) based on SLAM technology, to apply them to a wide range of sectors, including robotics [3], autonomous driving [4], and augmented reality [5]. These systems are rapidly emerging as versatile and accessible tools for 3D surveying, enabling the acquisition of complete models in significantly shorter timeframes, even in complex or hard-to-reach environments, and often incorporating Global Navigation Satellite System (GNSS) sensors for direct georeferencing [6]. SLAM-based systems offer features such as execution speed, completeness of the model, the ability to interpret materials, construction techniques, temporary or permanent works, the state of conservation, and any effects of past earthquakes on the structures. Moreover, estimating building elements’ dimensions, identifying load-bearing elements and visually inspecting them, and registering structural damage effects, as deformations and cracks, fits into the recent concept of twinning applied to built heritage, which offers, among other advantages, the possibility of sharing knowledge and working cooperatively even without direct access to the site under study [7].

The principle behind SLAM is the system’s ability to simultaneously build a map of the environment and locate itself within it in real time, using data from sensors such as LiDAR, inertial navigation systems (INUs) and, in some cases, RGB cameras [8]. Thanks to this approach, the device can track its trajectory in space even in the absence of a GNSS signal or external control points. The system combines spatial information collected by LiDAR with inertial and visual data (when present) to optimize position estimation and minimize drift errors accumulated during movement [9].

Among the main variants of SLAM-based portable systems, two fundamental approaches stand out: LiDAR SLAM and Visual SLAM, each with specific characteristics [10]. The LiDAR SLAM is based on the use of LiDAR sensors, which acquire the three-dimensional geometry of the environment through the direct measurement of distances. This approach guarantees good reliability in terms of spatial accuracy, even in variable or absent lighting conditions. Visual SLAM, on the other hand, relies on cameras (monocular, stereo or RGB-D) to extract visual characteristics and track movement over time. This type of system can offer additional information about the context, such as the color or texture of surfaces. However, its effectiveness is influenced by lighting conditions, the presence of texture-free surfaces, and phenomena such as reflections, which can compromise the quality of mapping. In some latest generation devices, hybrid approaches are frequently adopted, combining LiDAR and visual data, to improve the robustness and accuracy of the system even in complex or highly heterogeneous scenarios [11].

Although these systems do not reach the same levels of resolution and accuracy as DP and TLS, their use proves advantageous in contexts where operational efficiency is a primary concern [12].

In the field of cultural heritage, 3D surveys with SLAM-based portable systems have found application both in architectural survey [13,14] and in the archaeological field [15]. Recent studies explore their effectiveness in the 3D documentation of historic urban sites [16], underground environments [17], and excavation areas [3,18], where the operational speed and lightness of the devices constitute a significant advantage compared to traditional techniques. Thanks to the ability to perform dynamic, fast, and non-invasive surveys, combined with direct georeferencing supported by integrated sensors, these systems simplify the entire workflow, enabling efficient data acquisition and reducing time and costs.

In the context of architectural investigations, the potential of SLAM-based hand-held systems is a topic of growing interest among many researchers. Several studies have assessed the accuracy, reliability, and applicability of such systems in historical built heritage documentation [19]. Further contributions explored their performance in indoor and outdoor tasks [20] and their integration in digital heritage documentation strategies [6,21]. The analyses conducted demonstrate good consistency and reliability of the results, as compared to data acquired via TLS, which are often used as a reference [22].

TLS has been widely used for surveying historic buildings [23,24], also with the aim of supporting structural studies [25,26], while photogrammetric approaches, particularly those integrating UAVs and terrestrial photogrammetry, are increasingly widespread in practice, standing out for their flexibility, low costs, and high accessibility [27,28]. The use of UAVs significantly contributes to this accessibility, allowing efficient and safe data acquisition even in hard-to-reach areas [29]. Recent studies have further demonstrated the potential of advanced photogrammetric techniques for complex heritage scenarios, including the use of 360-degree cameras for efficient documentation in constrained environments [30] and the exploitation of photogrammetric models for detailed geometric and shape analysis of architecturally complex elements [31].

Despite the numerous advantages offered by TLS, such as the high precision and density of the data acquired, these tools present some intrinsic limitations that condition their use in certain operational contexts [32]. First, TLS is designed to operate in static mode, a condition that limits its use in situations where rapid data acquisition is required. Some TLS models are equipped with an integrated Inertial Measurement Unit (IMU), used for pre-alignment of scans. However, the accuracy of these IMUs is limited, and it can only be considered for an approximate solution, always requiring a post-processing for optimizing scan alignment. From an operational perspective, TLS requires careful planning. Due to the presence of occlusions and shaded areas, it is often necessary to carry out multiple scans from different locations to ensure complete coverage of the environment. This leads to an increase in survey times and greater complexity in the processing and recording phase. Finally, portability is also an aspect to consider. Although TLS were previously bulky and unsuitable for complex or difficult-to-access environments, increasingly compact and lighter models have been developed over time. These devices can also be mounted on photographic tripods, which are easier to handle than traditional topographic tripods, heavier and bulkier. This makes them more versatile, although in very complex contexts; nevertheless, despite this progress towards lighter and more flexible equipment, the SLAM-based hand-held systems seem promising [33], and extensive assessments of their performance in different operational contexts are highly advisable. This work aims to contribute to this purpose, focusing on efficient data acquisition and structural understanding [34].

To support multi-scale seismic vulnerability assessments of historical buildings, Sammartano et al. propose an integrated HBIM-GIS approach to enable a comprehensive spatial and structural analysis [35]. A 3D photogrammetric survey was used, along with a finite element analysis, to evaluate the structural behavior under seismic loads of the church of SS. Annunziata in Paestum [36].

In the context of 3D documentation of complex environments, particularly in the built heritage field, several studies have used residual distances between two 3D models to evaluate geometric coherence between point clouds acquired with different technologies [20,37]. Research such as [38,39] has highlighted how the cloud-to-cloud (C2C) comparison is a useful tool for estimating local deviations between datasets acquired via SLAM and TLS.

The present paper explores the potential of handheld and wearable SLAM-based scanners through a real-world application in the field of religious historical building studies, in the framework of a broader study addressing the safeguarding of small churches from a structural perspective, by further applying non-destructive diagnosis techniques [40] and FEM analysis.

Both qualitative and quantitative testing of the results is performed, based on ground truth and checkpoints. The operational specifics of SLAM-based systems are considered to assess their use in the field of built heritage documentation. A mix of indoor and outdoor scenarios is considered to critically evaluate the effectiveness of these devices and inform methodological choices in similar contexts.

Following metric considerations, the knowledge of the dimensions and geometric details of structural elements is a fundamental requirement for a reliable analytical analysis of their mechanical behavior [41]. Point clouds are also considered for morphological analysis of structural elements and to assess the degree of completeness of 3D models, supporting the development of simplified FEM models [42] and guiding preliminary diagnostic investigations. Data collected using SLAM-based systems supported numerical modelling, useful for predicting future structural behavior through specific simulations and analyses. Furthermore, these data were also used to calculate the vulnerability indices, as requested by Italian regulations [43] and as proposed by Lourenço [44].

## 3. Materials and Methods

This paper assesses the potential and limitations of SLAM technology in supporting preliminary structural analyses of parish churches in Lunigiana, a historical region located between Liguria and Tuscany in Italy. The parish churches, originally dating back to the 10th century, served as the main religious reference for the vast rural areas and exhibit some recurring architectural characteristics.

Many of them have undergone notable transformations over time, both due to wars and atmospheric agents, and due to the numerous earthquakes of varying intensity that have hit the Lunigiana region over the centuries.

This study considers two out of the more than 20 religious buildings identified as parish churches and spread across a vast territory. The case studies are identified based on their geometric and typological characteristics as representative: Pieve Santo Stefano in Sorano and Pieve di Santo Stefano in Vallecchia, both of which date back to at least the twelfth century and are mentioned for the first time in the papal bull of Eugene III in 1148. The two parishes, belonging to the ancient diocese of Luni [45], have undergone restoration, architectural changes, and natural degradation processes [46], making them ideal case studies to test the effectiveness of SLAM technology in supporting the specific requirements of the structural analyses being carried out as part of the broader research project. The work aims not only to verify the metric reliability of the acquired data but also to critically discuss the operational opportunities offered by these tools in the context of seismic risk assessment of cultural heritage churches.

### 3.1. Case Studies

#### 3.1.1. Pieve di Santo Stefano in Sorano

**Figure 1:**
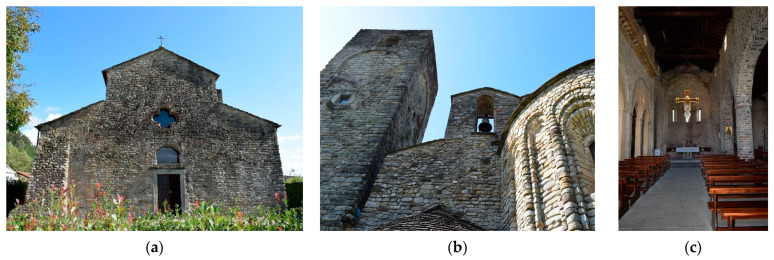
Pieve di Santo Stefano in Sorano: (**a**) facade; (**b**) apse and bell tower; (**c**) interior.

The Pieve di Santo Stefano in Sorano (Sorano Church) (Figure 1) is in the alluvial plain of the river Magra, near the village of Filattiera (Massa). The church stands on the remains of a previous pagan building, built by the Byzantines in the 6th–7th century, highlighting the sacred continuity of the site. The Romanesque building was built between the eleventh and twelfth centuries, and the current appearance of the church is the result of extensive restorations, concluded in 2000.

The structure retains its original layout, with a salient façade leading to an interior organized in three naves ending in three semicircular apses. The building features a load-bearing masonry structure made of river pebbles arranged in nearly regular courses. The façade features original openings subsequently infilled, resulting in masonry discontinuities that may be structurally significant. In the left corner, an external buttress is also present, introduced as a strengthening element to counteract thrust forces. Additional infilled openings are visible along the side elevations of the building, potentially affecting the continuity and uniformity of the masonry walls’ mechanical behaviour. On the rear elevation, a bell gable is present, structurally integrated into the perimeter masonry.

At the sides of the central nave large round arches resting on cylindrical stone pillars. In the late nineteenth century, the arcades of the left nave were closed off by pairs of arches and Neoclassical columns during the construction of family funerary chapels. Vaults, now no longer existing, were also built above each chapel, and the roofline was modified with the addition of a terracotta cornice. Numerous sculptural fragments and decorative elements, some of which have been reused in the current structure, attest to the richness of its original Romanesque decoration, such as capitals and reliefs with zoomorphic and plant motifs. These features highlight the church’s importance not only as a place of worship but also as a focal point of artistic and symbolic expression in the Middle Ages.

The square bell tower, likely constructed after the main body of the church, was originally conceived with a defensive function; its upper portion was characterized by large arched openings, which have since been infilled, resulting in alterations to the original structural configuration and stiffness distribution.

Historically, the church was part of a significant ecclesiastical and cultural hub, frequently visited by bishops and religious authorities, as evidenced by various epigraphic and documentary sources.

#### 3.1.2. Pieve di Santo Stefano in Vallecchia

**Figure 2:**
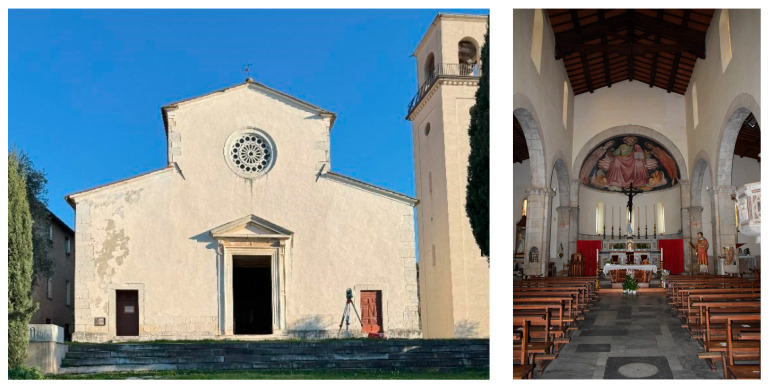
Pieve di Santo Stefano in Vallecchia.

The Pieve di Santo Stefano in Vallecchia (Vallecchia Church) (Figure 2), is located between Pietrasanta and Seravezza. Its first mention appears in a document from 881; later, in the Papal Bulls (1148, 1154, and 1203), it was named the parish church of Vallecchia. The church has undergone notable reconstructions and renovations over time; the wooden roof has been restored several times due to seismic events, war and natural decay.

The current structure was erected in the 12th century, using local marble, and features a basilica plan with three naves.

The masonry consists of “a sacco” technique, with well-squared ashlar blocks on the exterior with rock-fill. The three naves are limited by two rows of columns and pillars, enclosing an interior space that culminates in a semicircular apse, preceded by a triumphal arch and an elevated presbytery. The central nave is covered by a gable roof supported by three wooden trusses; similarly, the side aisles are roofed. The apse is topped by a hemispherical calotte, originally frescoed, and a terracotta roof covers the entire building.

On the counter façade, a painted wooden choir loft is supported by decorative corbels and houses a pipe organ built in 1881, which was later restored in 2004.

The façade, with its traditional gabled profile, is plastered but features a base and architectural detailing in squared blocks of local marble. The central portal, crowned by a triangular pediment, is flanked by two lateral entrances. A pierced marble rose window occupies the upper central part of the façade. Along the lateral walls, secondary entrances and numerous single-light windows (*monofore*) are present.

The bell tower, destroyed initially during World War II and rebuilt in more recent times, rises on the right side of the church. It features a square plan, plastered surfaces, and a belfry with four arched openings.

Significant restoration campaigns were undertaken between the 16th and 17th centuries, promoted by Ferdinando I de’ Medici and Maria Cristina of Lorraine. Further consolidation works were carried out in the post-war period.

The building exhibits a complex architectural stratification, combining elements of Romanesque, Renaissance, and Baroque styles.

### 3.2. The Acquisition Campaigns

**Figure 3:**
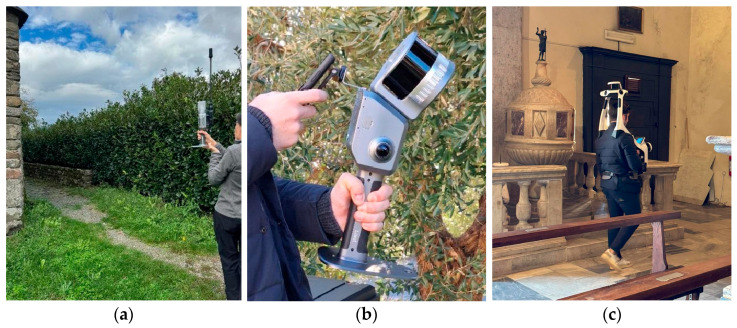
The tested instruments during the survey on the field: (**a**) Stonex X70GO with RTK module; (**b**) XGRIDS Lixel L2 Pro; (**c**) NavVis VLX 3.

A wide range of SLAM-based scanners are available nowadays; this work analyses data acquired using two hand-held and one wearable SLAM-based scanners: the X70GO by Stonex (New York, NY, USA) (Figure 3a), the Lixel L2 Pro by XGRIDS (Hong Kong, China) (Figure 3b), and the VLX 3 by NavVis (Munich, Germany) (Figure 3c).

Each tested SLAM system has specificities that affect its performance, versatility, and uptime. The main differences concern the physical configuration of the instrument (handheld or wearable), the type and number of LiDAR sensors, cameras, the RTK module for georeferencing, and the acquisition modes. Table 1 summarizes these differences, providing a comparative overview useful in assessing the strengths and limitations of each system.

Regarding the possibility of direct georeferencing, the Lixel L2 Pro integrates an RTK receiver, however RTK functionality was not activated during this study. An external module can be added to the X70GO; however, no GNSS options are available when working with the NavVis system.

The scan rate, the implementation of visual SLAM, and the integration with RTK GNSS systems affect performance and scope of use. Some models focus more on visual quality, with high-resolution cameras, like Lixel L2 Pro, while others declare to equally value accuracy, like VLX 3.

As shown in Table 1, the VLX 3 stands out for its high scan rate; the Lixel L2 Pro offers a fairly good resolution, although its point distribution is less dense than that of the VLX 3, while the X70GO has a lower resolution. The resolution of the final 3D model clearly depends heavily on the acquisition speed. However, other factors also play a significant role, such as the speed at which the track is travelled and the geometry of the track itself. It is also important to consider that during data processing, the option of oversampling is becoming increasingly common. This elaboration obviously favors qualitative aspects such as data visualization and interpretation, while it does not affect quantitative aspects such as accuracy.

All the instruments allow for viewing the acquired data in real time via a mobile phone connected to the device, thus supporting the operator in quickly checking any gaps in the area coverage being surveyed.

An option provided by the X70GO is to operate in static mode, standing on a specific single-legged stand and allowing for the acquisition of denser and more detailed point clouds through the so-called XWHIZZ scans; however, this mode was not analyzed in the present study.

The survey campaign was carried out over two days, one in November 2024 and one in January 2025, and included both interior and exterior scanning operations.

Data acquired with SLAM technologies are commonly validated by comparison with measurements carried out using a TLS [12,21,37]. In this study, however, a topographic survey with a total station was used as ground truth for assessing the accuracy of SLAM models. Total station surveying provides data which, although low density, is reasonably accurate and is not affected by any residual alignment errors in TLS scans, making it a robust benchmark for evaluating SLAM-derived models.

**Figure 4:**
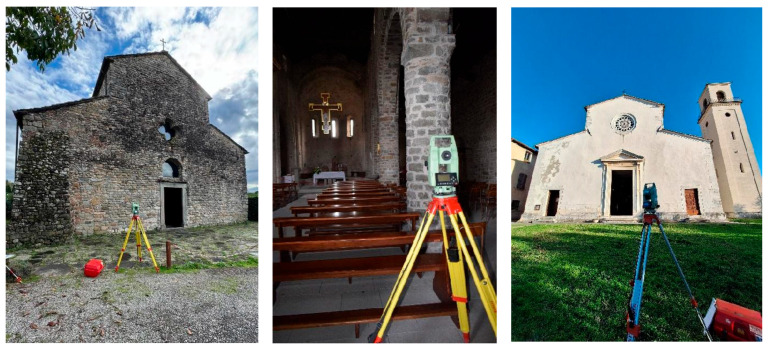
Total station surveying: Sorano Church (**left** and **centre**) and Vallecchia Church (**right**).

Two topographic vertices had materialized (Figure 4), one located outside and the other inside the church. From these reference points, a series of target and natural points were measured, strategically distributed throughout the site. These included points evenly distributed on the facade, counter-facade, side aisles, floor, and ceiling, providing a comprehensive framework for the geometric verification of the SLAM-based point clouds.

All three SLAM devices have been used for measurements in both indoor and outdoor environments, following closed trajectories (looped paths) that begin and end at the same point: this is a key strategy to reduce drift errors and increase the geometric consistency of the dataset. Moreover, when working without the RTK module, Lixel L2 Pro mandatorily requires starting and ending points to coincide to ensure the reliability of the dataset.

Although all devices allow the survey to be carried out by a single operator, for practical and organizational reasons, the survey was conducted by two operators. This choice made it easier to manage the equipment and follow the planned route. The acquisition time was between 6 and 25 min, depending on the instrument used and the extent of the detected area.

**Figure 5:**
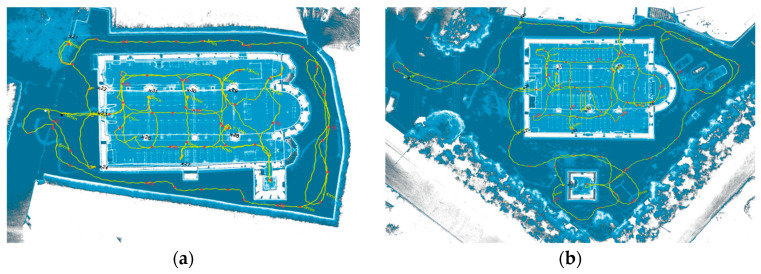
Path followed during acquisition with VLX 3: (**a**) Sorano Church and (**b**) Vallecchia Church.

For the VLX 3 (Figure 5) and Lixel L2 Pro instruments, the acquisition paths were intended to digitize the interior and exterior of the entire church (and the bell tower as well) in one go. The Lixel L2 Pro device was used exclusively for the Vallecchia Church dataset, following the same acquisition path as the VLX 3.

**Figure 6:**
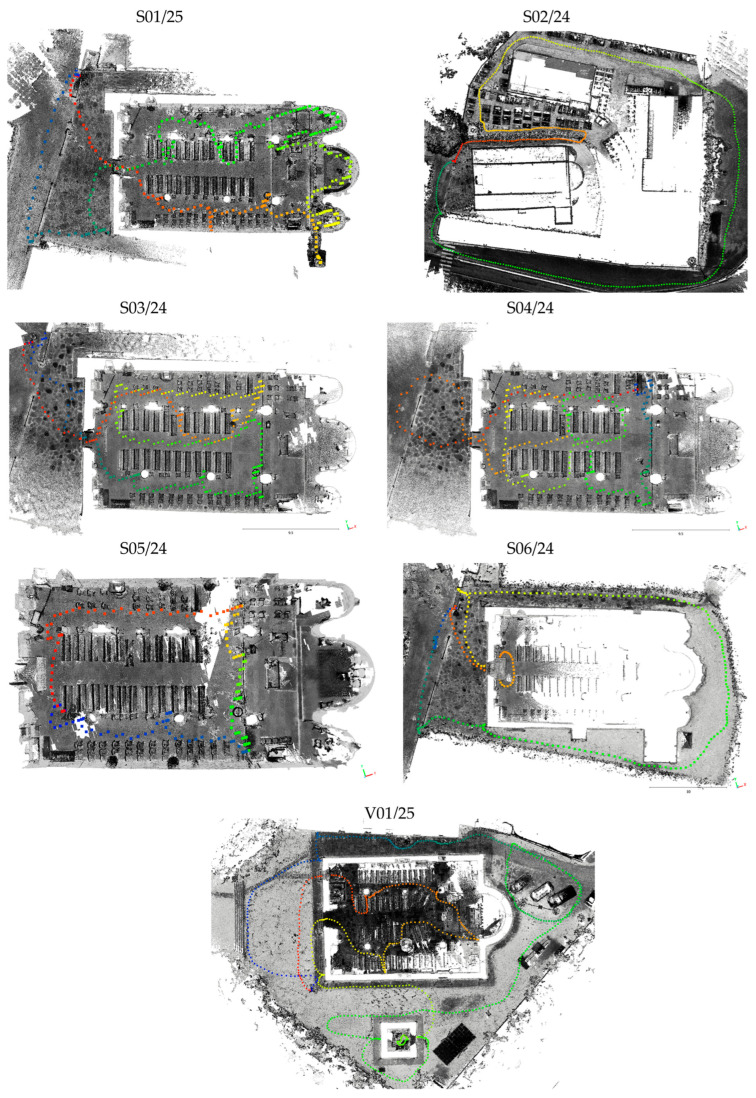
Paths followed during acquisition with X70GO: Sorano Church (S01/25, S02/24, S03/24, S04/24, S05/24, S06/24) and Vallecchia Church (V01/25). Colors from blue to red express the acquisition time.

With the X70GO, on the other hand, several scans were carried out (Figure 6), the main objective being to test how path geometry and Ground Control Point (GCP) availability affect the final 3D model, as discussed in Section 4.1.

The X70GO requires a critical initialization phase, during which the SLAM algorithm calibrates the initial position and begins to build the map. For proper operation, it is essential to avoid the presence of moving objects in the sensor’s field of view. The sensor must be placed in a stable position and left still for approximately one and a half minutes.

Similarly, the Lixel L2 Pro also features an initial calibration phase, which lasts about one minute.

In contrast, the initialization and finalization phases of the VLX 3 are characterized by the acquisition of a marker: the operator must stand on and register such a point at the beginning and end of the survey to ensure the closure of the SLAM trajectory and a proper alignment of 3D data.

#### The Off-Line Processing

In the present work, SLAM-based hand-held systems are analysed and compared, with particular attention to the data post-processing phase. This phase was carried out using proprietary software specific to each device: GOpost for X70GO, NavVis IVION for VLX 3, and LixelStudio for Lixel L2 Pro (Table 2).

The software used, in addition to reconstructing the 3D scene, offers advanced features that significantly influence the result, such as point cloud optimisation, automatic removal of dynamic objects, upsampling, and management of 360° panoramic images. However, the software has limited technical documentation and offers few options to adjust or optimize parameters, which restricts control over the results. All the producers provide user-friendly solutions, which are highly effective for professionals. On the other hand, this makes it challenging to recognise how the final model is affected by raw data (i.e., effects related to sensors) or by post-processing (i.e., effects related to software).

The differences in sensors, workflows, and above all in the software solutions adopted place limits on a purely quantitative comparative analysis. However, through the observation of available functionalities, the results obtained, and the limitations identified, it is possible to outline the strengths and critical points of each solution.

A crucial aspect that emerged during processing concerns the resolution of the acquired data. As will be explored in depth in the following section, the resolution obtainable through tools based on SLAM technology is borderline for applications in the built heritage documentation.

The relationship between geometric resolution and texture resolution assumes particular importance: a denser point cloud does not always guarantee a more effective representation, due to noise effect, and the availability of high-definition images play a key role, considering that the overall interpretation of the 3D model depends not only on the point density but also on the quality and coherence of the textures applied.

The tested software offers different solutions to address this problem: some make use of automatic densification functions; others propose controlled resampling techniques. However, as discussed in the further, these approaches are partially effective, since the increase in density does not necessarily lead to a significant improvement in the readability of architectural details on the recorded data.

Promising perspectives also emerge from innovative approaches, such as Gaussian Splatting, which exploit panoramic images and camera positions to build immersive 3D representations. To this end, MMSs are used to define the external orientation of images, thereby reinterpreting the SLAM paradigm, which was initially developed for the simultaneous solution of localisation and mapping problems. Localisation then becomes instrumental in the production of the map. Finally, the map (as a point model) becomes almost negligible compared to the Gaussian Splatting derived from the localisation of the cameras, whose orientation is defined precisely through “localisation”.

The post-processing phase, for all software analysed, took between one and five hours for each path detected, depending on the survey duration, the environment complexity, and the texture calculation, included in the elaboration time (Table 3). The data processing time of VLX 3 and Lixel L2 Pro is higher, as the routes include both survey of the exterior and interior of the church, while the X70GO datasets include shorter paths (Table 4).

LixelStudio for Lixel L2 Pro provides advanced tools for point cloud optimization: it is possible to automatically remove dynamic objects, thus improving the cleanliness of the scene and reducing interference caused by the presence of moving elements. Furthermore, it is possible to carry out resampling (specifically, oversampling), to obtain a uniform and regular point cloud, and therefore a more defined texture. In our case, a 5 mm pitch has been adopted; however, the system allows for a resolution of up to 1 mm to be achieved. The NavVis IVION software, used for VLX 3 data processing, also allows defining the resolution of the point cloud, with a resolution that can reach up to 5 mm (adopted value). The 3D model shows some relevant differences in point density, as better described in Section 4.3.

In the GOpost software for X70GO, the workflow consists of calculating the cloud from SLAM data, then optimizing it with a noise-reduction filter. In this step, there is an option for “densification”, an oversampling method that depends on the resolution of the original cloud and produces a model three times denser. In the software version adopted for the data processing presented here (November/December 2024), this option was not optional.

NavVis IVION enable the automatic blurring of people’s faces and plates on the texture, a key aspect when privacy protection is crucial, while GOpost and LixelStudio includes automatic removal of people and moving objects.

In addition to these features, both LixelStudio and NavVis IVION support the generation and management of 360° panoramic images. These panoramas are captured during the scan and provide an immersive visual context of the environment, which can be navigated interactively and used to support the interpretation of the point cloud.

**Figure 7:**
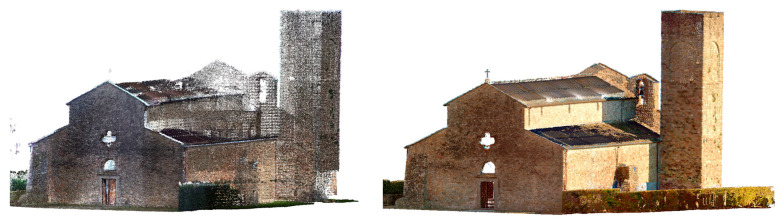
Sorano Church: data acquired respectively with the X70GO and VLX 3.

**Figure 8:**
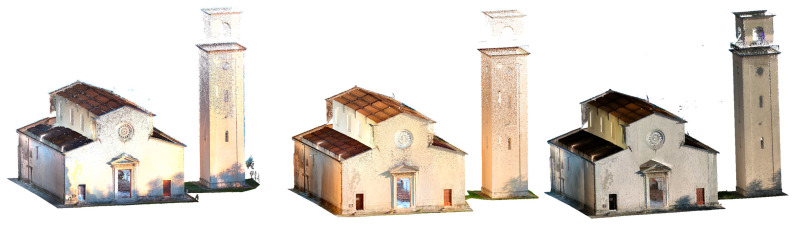
Vallecchia Church: data acquired respectively with the X70GO, VLX 3 and Lixel L2 Pro.

Figure 7 and Figure 8 show a view of the final data acquisition result.

## 4. Results

Until relatively recently, SLAM-based systems were generally considered borderline technology for applications requiring a high level of detail, due to limitations in sensor quality and algorithmic maturity [47]. However, recent advancements in both hardware and software have significantly improved their performance. Today, SLAM is increasingly competitive with well-established techniques such as TLS, particularly in terms of resolution and ease of use [48]. At the same time, accuracy, although improved compared to the first products marketed, continues to be an aspect that is not always appropriate in many applications.

This section examines and evaluates the 3D point clouds generated by the SLAM-based system through both quantitative and qualitative analyses. As a first point, it is worth mentioning how drift effects can affect the trajectory, suggesting the use of looped paths and GCPs to strengthen it. GCPs, at the same time, allow for georeferencing [19] or at least to reference data in a common reference system in case of control network measured by total station.

In the case studies, the georeferencing of the point clouds has been carried out using GNSS data acquired during the SLAM survey conducted on both churches. At the same time, several targets placed on the site (inside and outside) have been measured with the total station. To also bring datasets recorded without GNSS (but with TS surveyed targets) into the same reference system, one point cloud was referenced based on the GNSS and on the TS target as well, and then aligned each other (fixing the GNSS-based dataset as the reference); the alignment was performed in CloudCompare 2.13 [49] software and optimized with the Iterative Closest Point (ICP) algorithm. The resulting transformation matrices were then applied to translate and rotate the GCPs acquired during the topographic survey into the global reference system, enabling the georeferencing of the dataset acquired without GNSS support as well.

### 4.1. Analysis of Georeferencing Accuracy

To evaluate the influence of the GCPs configuration on the accuracy of georeferencing, acquisitions with different characteristics were carried out.

Several datasets have been acquired by the X70GO, starting both inside and outside the churches, and recording data indoor and/or outdoor. Lixel L2 Pro and VLX 3 always start outside and scan the buildings, both indoors and outdoors, with a single, closed path.

The paths followed for data acquisition are illustrated in Figure 5 and Figure 6; the use of GPS and the number of targets are summarized in Table 4. It summarizes the datasets acquired on both churches, with different instruments and georeferencing strategies: some have been georeferenced by GNSS, while others by targets. Even though all targets available along the trajectories were captured during all acquisitions, when processing data considering GNSS support, the software does not provide residuals on targets. Therefore, in order to evaluate the contribution of the RTK module, the models obtained with GNSS-supported georeferencing and those with target-supported georeferencing were compared (Section 4.2).

In the processing of some datasets acquired with X70GO, the orientation of the point cloud was carried out by using some targets as check points (CPs) to better evaluate the trajectory accuracy (S03/24 and S01/25 Sorano Church; V01/25, Vallecchia Church). The georeferencing accuracy assessment was conducted through the residuals of targets which have been independently measured, considering planimetric residual and those along vertical direction (Table 5).

In both case studies, around ten targets were distributed across the scene, all of which were initially used in the computation. The average planimetric residuals are about one centimeter, with RMSE between 12 and 15 mm. The highest values at the 95th percentile are around 3 cm. No bias on vertical direction, as demonstrated by all the mean values around 0.

The paths S04/24 and S05/24 in Sorano Church are wholly or almost indoor: they cover the same area, the former following a more articulated path, the latter a linear closed path. The horizontal and vertical residual distances, although calculated only with respect to the GCPs, are completely negligible.

In a second series of processes, only those pathways that had acquired almost all the available targets were considered, so that half of them could be kept as CPs. The statistical parameters referring to the CPs are very close to those characterizing the GCPs, with differences of less than one centimeter. In the last path (Vallecchia Church V01/25), one of the targets (considered as CP in the second processing) shows a planimetric residual of approximately 3 cm, suggesting an outlier in the topographic measurements, as evidenced by larger deviations and a higher dispersion, despite similar maximum values.

### 4.2. Analysis of 3D Models Accuracy

As already mentioned, the accuracy evaluation of the acquired dataset is performed by referencing to a ground truth made by a sparse point cloud measured by total station. Due to the reference dataset having a very different density from that of the datasets to be tested, the nearest-neighbor distance would yield incorrect results [50,51]: in fact, for each point of the tested cloud, the algorithm implemented in CloudCompare searches the nearest point in the reference cloud and computes the Euclidean distance, which in these conditions can be influenced more by the different spacing between the points than by the distance between the models. However, the software offers the possibility of defining a shape model, albeit very approximate, with respect to which the distance from the reference is calculated for each point in the test dataset, thus providing reliable results [52].

**Figure 9:**
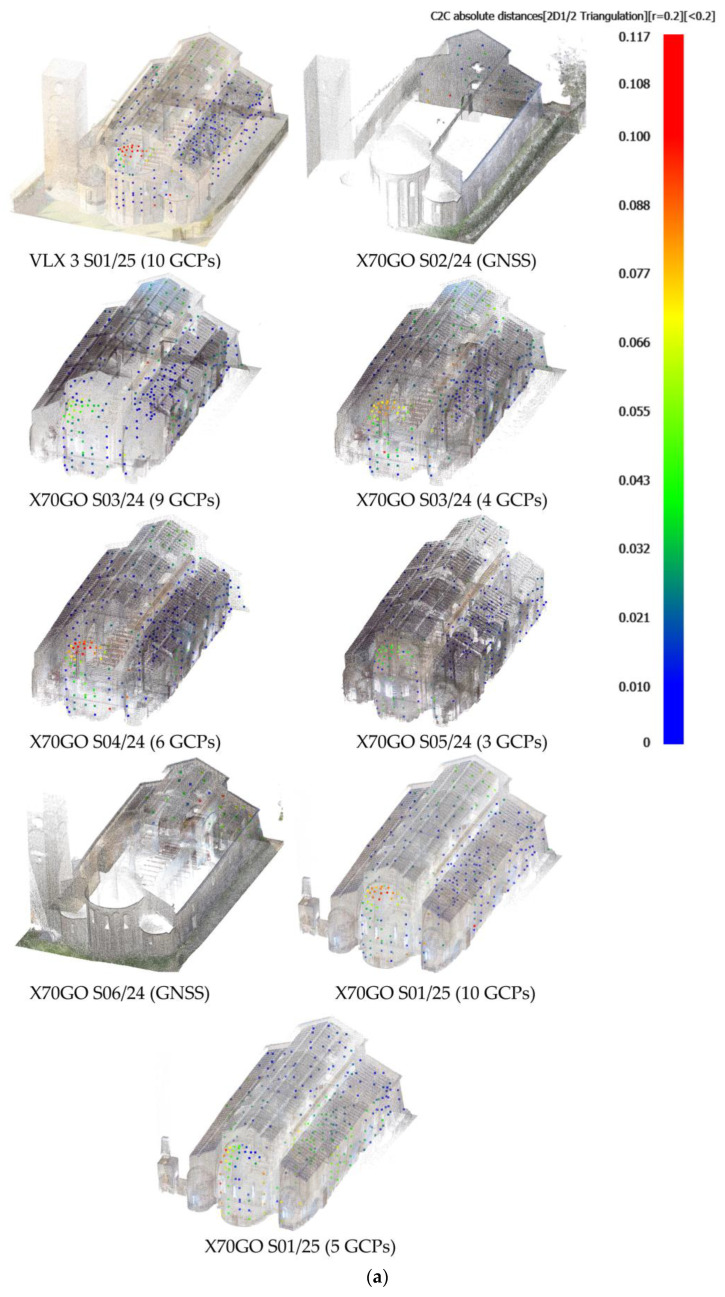

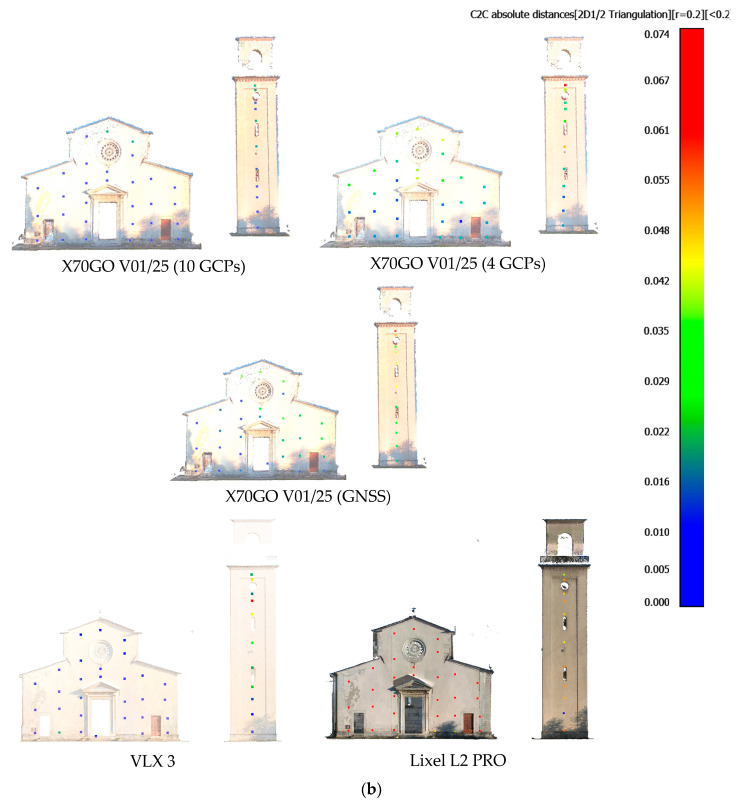
Residual distances between SLAM-based point cloud and the topographic survey: (**a**) Sorano Church and (**b**) Vallecchia Church.

Each point is colored based on the computed distance, adopting a color ramp from blue (minimal discrepancies) to red (larger discrepancies) [53] (Figure 9).

Also here, comparisons are made considering both datasets aligned with all available targets and those with a reduced number of targets, as well as those referenced with GNSS. The numerical results of these comparisons are presented in Table 6, while Figure 9 shows the distribution of the 3D residual distances across the datasets considered. In Sorano Church, the reference topographical data are distributed both inside the building and on its façade, while in Vallecchia Church, they are only on the façade and bell tower.

The Sorano church presents systematically significant residues in the apse, an aspect that deserves further investigation, given the simple geometry and limited acquisition distances. For those points, the Z component shows mean values smaller than 1 cm, but XY components reach up to ten centimeters. This could be related to the topographic measurements, carried out for those points according to very foreshortened lines of sight.

In general, the maps show a slight increase in residues in the upper parts of the churches, which is more evident on the outside (on the façade) than on the inside, where the geometry available for reconstructing the scene is more complex. The comparison between the performance of the three instruments used is highlighted by the results on the facade of Vallecchia Church: homogeneous and limited residues for the VLX 3 dataset, partly affected by the scene’s geometry for the X70GO, and on average higher for the data collected with Lixel L2 Pro. Table 6 shows the relevant statistics, distinguishing between the planimetric and altimetric components.

In both case studies, when the same route was processed with both targets and GNSS, the latter solution produced slightly worse results, demonstrating the better accuracy provided by georeferencing supported by a topographic survey compared to the GNSS option. However, it is important to consider that this approach is more streamlined in the field and significantly reduces working times. At the same time, it must be taken into account that the churches covered by this study require most, if not all, of the trajectories to be indoors, which means that GNSS support is lost. In paths that are partly indoor and partly outdoor, the starting point was always set outdoors; in all cases, there were difficulties in reconnecting to the GNSS signal once back outside after travelling indoors. The idea of starting the survey outside, then proceeding inside the building and finally concluding with a further outdoor route was tested in route S01/25 (Sorano Church), which, however, presents the worst results among the acquisitions with X70GO. This approach obviously solves the need to georeference the indoor survey, but it compromises the accuracy of the model to some extent.

About the reduction in GCPs, from about ten to about half, carried out in the processing of some routes (S03/24, S01/25 in Sorano Church and V01/25 in Vallecchia Church), the results of the comparison with the reference dataset show high stability, both in the first two routes, which are mainly indoor and have a high overlap of data recorded during acquisition, and in the last one, which has also a significant outdoor section. In all these cases, the residual planimetric distances have a median between 5 mm and 12 mm, and at the 95th percentile, the values do not exceed 5 cm.

Comparing the three instruments tested, the VLX 3 achieved the best results. It should be noted that, in both churches, the survey was carried out with highly redundant trajectories, defining multiple loops. Lixel L2 Pro, on the other hand, achieved the worst results, showing significant planimetric residuals, although the altimetric residuals are minimal, as for the other two instruments.

The X70 scanner, tested more thoroughly with the acquisition of redundant scans in the case of Sorano Church, when used with the RTK module, has a median just above 2 cm in only one case and values above 5 cm at the 95th percentile (S01/25 in Sorano Church, already discussed above); considering target-based referencing, the median falls below 1.5 cm, but at the 95th percentile, the values are still in the order of 5 cm.

Only one point cloud was acquired by the Lixel L2 Pro; the residues in that dataset have the highest values, reaching the order of the decimeter.

The results indicate that the derived geometry is sufficiently accurate to extract the data required for the computation of simplified vulnerability indices [44], for which it is essential to know the surface areas of load-bearing walls in the x and y directions, the heights and plans of structural elements such as walls and columns, and the total weight of the structure. Regarding the index based on the analysis of collapse mechanisms [43], one key requirement is knowledge of masonry slenderness, which can be easily assessed using 3D models by comparing them with vertical reference surfaces.

### 4.3. Analysis of 3D Model Density and Resolution

Point cloud density is a critical factor in determining the ability to read fine details in the point cloud: too low a density results in striping artifacts and small features may be missed. Some methods are proposed to determine the “appropriate point cloud density” (as the point at which the data quality can no longer be significantly improved by increasing the point cloud density, all other conditions being held constant), as stated in [54] where airborne LiDAR point cloud are considered. The huge variability of conditions in the field of built heritage generally leads to the optimal sampling rate being defined on an empirical basis, as also suggested in [55]. Moreover, as mentioned in “The Off-Line Processing”, commercial software that processes the sensor data tested in this work frequently oversamples the points.

While “density” represents how dense the points are in space, and is therefore an objective data, “resolution” is related to the level of detail that can be distinguished and is therefore affected by a subjective component.

The following paragraphs, therefore, propose a separate assessment of these two aspects.

#### 4.3.1. Point Cloud Density

The purpose of this analysis is to evaluate the quality of the obtained scans from a local-scale perspective, considering the point clouds exported by the processing software, avoiding oversampling whenever possible.

The density values were calculated locally on selected test areas, with the aim of evaluating the ability of the system to correctly reconstruct all the geometric characteristics of the documented spaces.

To estimate point cloud density, the precise density computation procedure offered by CloudCompare is applied, giving as a result the count of points within a defined neighborhood around a central point.

**Figure 10:**
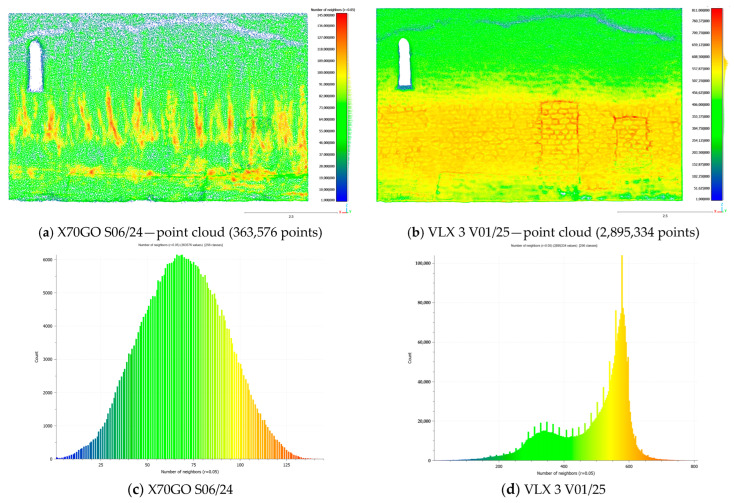
Density evaluation results: Sorano Church—external right wall.

The left wall of the Sorano Church was initially analyzed, both for the X70GO dataset and for the VLX 3 dataset, since the acquisition path in both cases is very similar and the dataset can be considered as acquired from a single “run”. (Figure 10). As mentioned before, both datasets during the processing underwent a resampling process to regularize and densify the data, with minimal room for operator intervention. Despite this, there are clear differences in the amount of data referring to the same portion of the building, although these are also obviously influenced by other parameters, such as acquisition distance and travel speed. For this reason, the two maps and their histograms cannot be directly compared. However, when considered individually, they show that the data from VLX 3 is mainly affected by the acquisition distance (Figure 10b): in fact, it has a higher density at human height, where the sensor-wall distance is minimal, and increases towards both the floor and the ceiling as this distance increases; the histogram (Figure 10d) shows a bimodal distribution. The dataset from X70 (Figure 10a), on the other hand, appears to be influenced by the acquisition speed, or more specifically, by variations in this speed.

**Figure 11:**
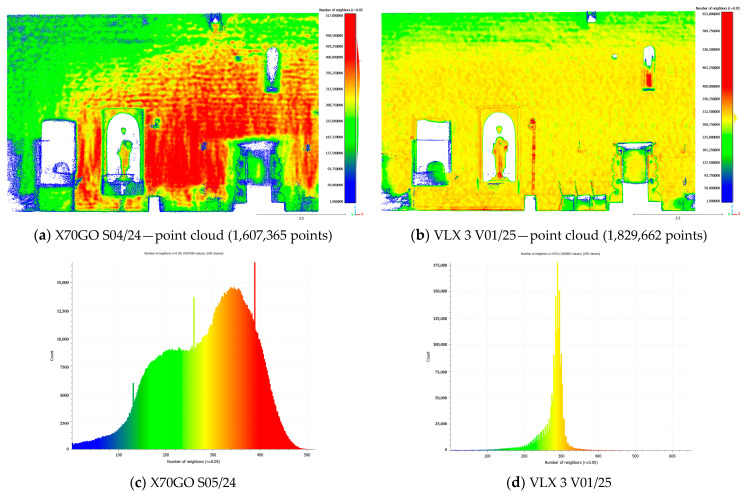
Density evaluation results: Sorano Church—internal left wall: (**a**) X70GO data, acquired in the path S04/24; (**b**) VLX 3 data, acquired in the path V01/25. The graphs (**c**) and (**d**) show the number of neighborhood points within a 5 cm radius sphere: the less dispersed the Gaussian distribution, the more homogeneous the point density.

Subsequently, the left inner wall of the church was also analyzed for both datasets; the area was scanned multiple times with both instruments, following homogeneous acquisition paths, obtaining a similar total number of points in the X70GO and VLX 3 datasets (Figure 11). This time, the bimodal distribution is what makes the X70GO histogram stand out, showing how scanning the same area multiple times (in this case, the part of the wall on the right in Figure 11c) affects it. In this case, the NavVis dataset presents a much more concentrated distribution: indeed, the map shows an almost uniform distribution, reduced only at the top of the wall (Figure 11b).

As expected, the analysis of SLAM point cloud density reveals a direct correlation between density values and the distance from the sensor to the scanned surface. This relationship is influenced by the operator’s trajectory and the amount of time spent scanning specific areas.

The point cloud acquired with VLX 3 exhibits a more homogeneous density distribution (Figure 11d) compared to the X70GO dataset (Figure 11c). In the VLX 3 case, lower density values are found only in the upper portions of the wall, likely due to the increased distance between the sensor and those areas during acquisition. Conversely, the X70GO dataset shows greater variability in density. Resampling applied during data processing can strongly affect the resulting point models.

Similar considerations come taking into account the surface density parameter instead of the number of neighbors. In general, cloud density varies and it does not consider the distribution of points on the object surfaces. As a result, it is not an exhaustive parameter for expressing the quality of a model, although it is a key factor for 3D data interpretation.

#### 4.3.2. Point Cloud Resolution

To further study the ability of different SLAM systems to capture complex architectural features, a detailed analysis was conducted on selected portions of the churches, where significant structural and decorative elements are present, thus enabling a more accurate assessment of the system’s ability to document geometry and surface details, particularly about structural evaluation and heritage conservation needs. Across all datasets, architectural details are more clearly discernible when surfaces are scanned from multiple angles, highlighting the importance of the acquisition strategy. For structural studies, the model must accurately represent discontinuities and defects, as well as model arcs, vaults, and other load-bearing elements that influence load distribution.

**Figure 12:**
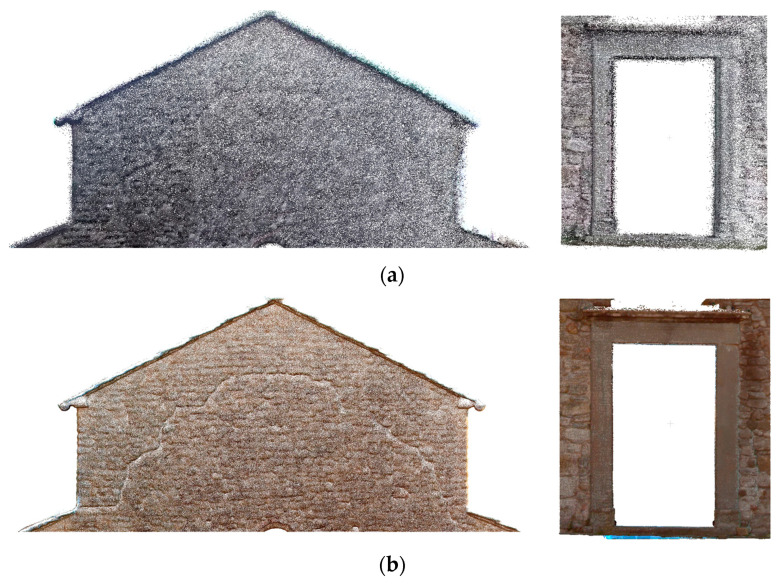
Sorano Church: point cloud of the top part of the facade and detail of the entrance door: (**a**) X70GO (435,576 points and 155,504 points) and (**b**) VLX 3 (1,149,693 points and 694,997 points).

**Figure 13:**
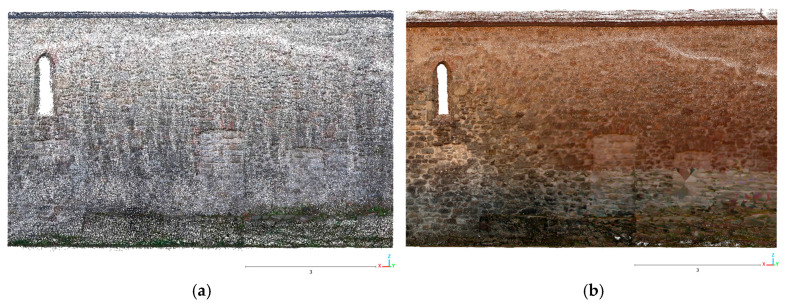
Sorano Church: left wall point cloud: (**a**) X70GO (467,387 points) and (**b**) VLX 3 (3,675,141 points).

**Figure 14:**
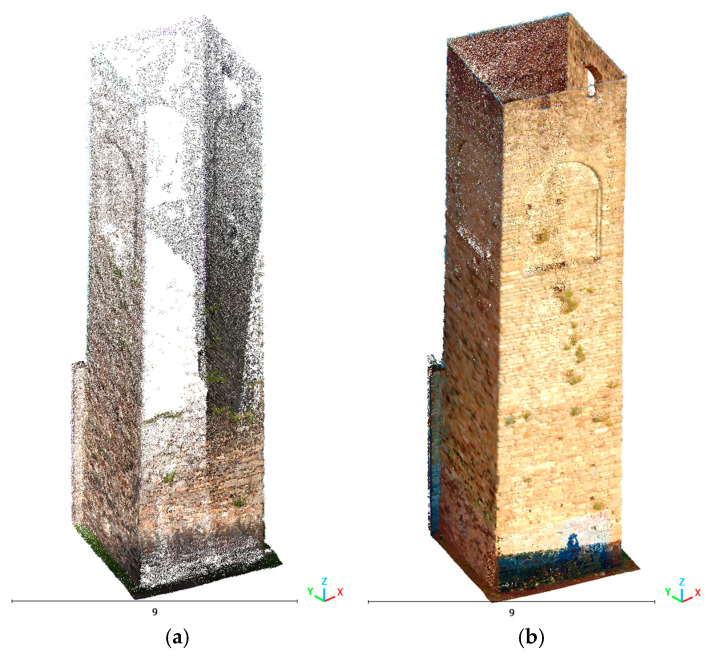
Sorano Church: points cloud of the bell tower: (**a**) X70GO (538,570 points) and (**b**) VLX 3 (5,670,750 points).

At the architectural scale, the point cloud generated by the X70GO system does not capture geometric features such as wall texture or architectural details (Figure 12, Figure 13 and Figure 14). Nevertheless, the overall data quality is sufficient to support architectural representations enabling structural evaluations relevant to restoration and consolidation planning.

In contrast, the higher density of the VLX 3 dataset allows for a more detailed examination of architectural elements (Figure 12, Figure 13 and Figure 14). Although density and, therefore, readability tend to decrease with distance (and, therefore, elevation), the VLX 3 system maintains a higher level of quality in the upper portions of the structure compared to the X70GO, providing more reliable data for documentation and analysis even at greater heights. In the VLX 3 datasets, the original facade (Figure 12), the traces of previous openings on the left side of the church (Figure 13) and the bell tower (Figure 14) clearly register the variety of materials used, as evidence of the different phases of renovation that the building has undergone.

**Figure 15:**
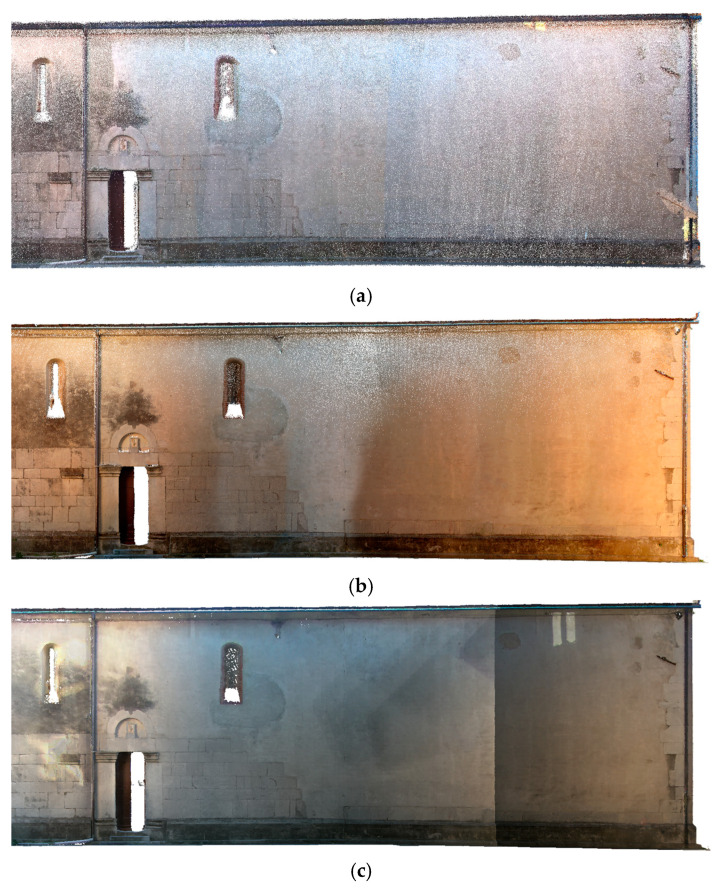
Vallecchia Church: point cloud of the left side elevation of the church: (**a**) X70GO (3,275,499 points), (**b**) VLX 3 (5,827,879 points), and (**c**) Lixel L2 Pro (9,773,859 points).

**Figure 16:**
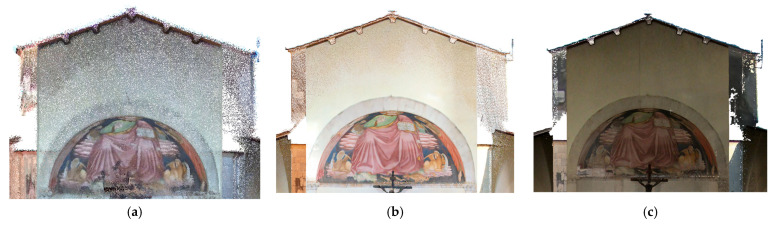
Vallecchia Church: point cloud of the tympanum of the triumphal arch: (**a**) X70GO (834,096 points), (**b**) VLX 3 (3,141,418 points), and (**c**) Lixel L2 Pro (6,747,285 points).

**Figure 17:**
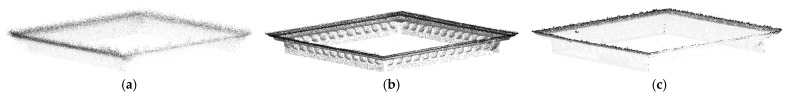
Vallecchia Church: point cloud of the bell tower frame: (**a**) X70GO (62,051 points), (**b**) VLX 3 (126,674 points), and (**c**) Lixel L2 Pro (818,727 points).

The higher point density of both VLX 3 and Lixel L2 Pro (Figure 15, Figure 16 and Figure 17) allows for a more detailed reading of architectural features, which become significantly more legible when scanned from multiple angles. However, readability tends to decrease with height, due to the increased distance between the sensor and the scanned surfaces. Among the tested systems, VLX 3 demonstrates the highest consistency in data quality at longer distances, outperforming both Lixel L2 Pro and X70GO in the acquisition of upper-level details. Despite having a lower point density than the L2PRO dataset, the VLX3 acquisition exhibits superior visual interpretability of architectural details. This improved readability is attributed to higher local geometric consistency and reduced spatial noise, which enhance the definition of edges, surface continuity, and decorative elements. Conversely, the higher point density of the L2PRO data tends to amplify residual SLAM misalignments and measurement noise, resulting in thicker surfaces and less distinct fine details. These results indicate that point density alone is not a reliable indicator of data quality for detailed architectural analysis.

The type and quality of the data may directly affect the structural reading. The detailed point clouds allow for the analysis of the irregularities of historical walls, highlighting elements such as misalignments, changes in thickness, or anomalies. For example, if high-resolution 3D data can capture the morphology of cracks or fissures in masonry, it is possible to map their extent, width, and orientation. If the tower is slightly sloping, skewing, or warped outside the lead, an effective 3D point model allows these anomalies to be identified precisely.

The comparison between the instruments illustrates the significant impact of data density and resolution on the reliability of the evaluations. Considering the texturized 3D model appearance, the Lixel L2 Pro software’s upsampling techniques offer a considerable advantage, ensuring greater uniformity in the point cloud in a way similar to what researchers describe in [56]. However, the increase in density does not automatically translate into an increase in the level of architectural detail and it does not necessarily add descriptive valuable information to the reading of architectural elements. In the VLX 3 model (Figure 13b and Figure 14b), despite the greater density of the point cloud, some texturing problems are highlighted, particularly in the lower part of the wall, related to variations in lighting during acquisition and insufficient photographic coverage by of the instrument.

The interpretation of decay and structural irregularities to support seismic vulnerability analyses and conservation strategies is not always immediate or clearly visible, particularly in datasets acquired with the X70GO system. Surface irregularities, cracks, and material loss can be identified and, albeit in a summary manner, quantified directly in the point cloud acquired with VLX 3, thanks in part to the integration of colour information and intensity values.

The integration of information on degradation and structural irregularities into numerical models enables a more accurate representation of the building’s actual state. Point clouds, therefore, demonstrate a non-invasive and visually supportive tool for the joint assessment of the geometry and degradation of structures.

### 4.4. Analysis of 3D Model Completeness

Capturing a complete building point cloud is a challenging task in most real-world situations. Techniques have been developed to make effective the digitization of small objects, generally using robotic systems that move the object and/or sensor to optimize their relative positions [57]. With drone photogrammetry, it is possible to set a flight plan based on a coarse model of the object being surveyed to limit lacunae in the 3D model as much as possible [58], and deep learning techniques can refine poor textured surfaces in image-based digitization projects [59]. Advanced reconstruction techniques based on geometric primitives, such as those proposed in [60], offer a tool for improving the continuity and integrity of 3D models by exploiting the presence of regular surfaces to complete mesh surfaces, thus facilitating structural interpretation and simplification of computational models. Generative approach [61] and data-driven methods have also shown promising results in this area: ref. [62] proposes a neural network-based approach for archaeological artefacts shape completion and virtual restoration, which would be promisingly extended to broader fields of study.

In general, however, for a building 3D survey with either TLS or DP, constraints come from the conformation of the site, the characteristics of the instruments, and the need to operate both indoors and outdoors, which means that some parts of the object are not recorded. An incomplete surface coverage entails missing feature detection and demanding post-processing. It is also not easy to assess how complete a point model is, as the element of comparison should be the object itself [59]. Several solutions have been developed for small object and artifact digitization, and no-reference methods are proposed to assess the point cloud quality [63].

To assess the geometric completeness of the digital acquisition of the church in Vallecchia and its bell tower, a metric comparison was conducted between an ideal reconstructed 3D model and the point clouds. In particular, a threshold in a cloud-to-cloud distance analysis was set to assess the point model completeness, assuming that the portions of the reconstructed model that are beyond the set threshold correspond to ‘missing’ data in the point model [64]. The object geometry has been reconstructed, disregarding minor details, and, depending on the object tested over time, based on geometrical and structural assumptions. The reconstructed surfaces, generated by profile extrusion, have then been transformed into point clouds, with a resolution comparable to that of the models to be tested.

**Figure 18:**
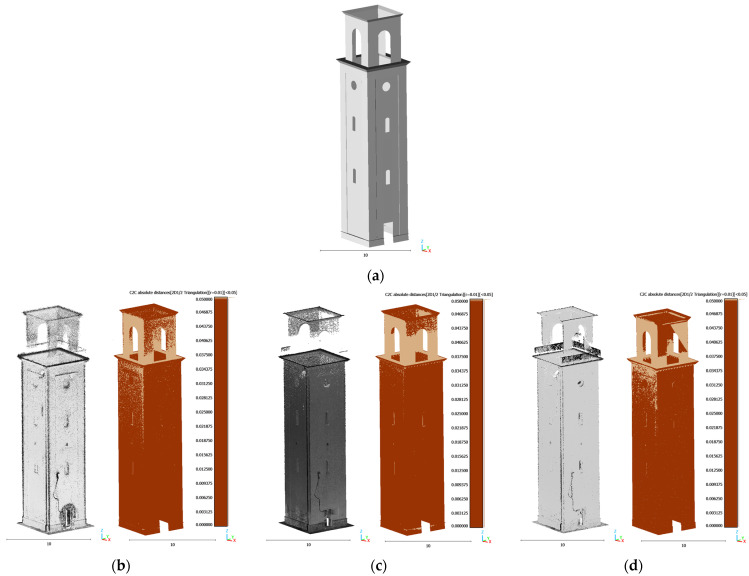
Completeness assessment of the bell tower of Vallecchia Church point clouds: (**a**) reference model (**b**) X70GO, (**c**) VLX 3 and (**d**) Lixel L2 Pro.

The C2C comparison was performed using the reconstructed model as the reference, while the point clouds served as the test datasets. The results are visualized through a color-coded map of absolute distances, with a maximum threshold set at 5 cm. Considering that points in the reference (and complete) 3D model farther than the threshold didn’t have any correspondence, the corresponding area on the test model (the SLAM-based) is missing. Points beyond this threshold were highlighted in pale brown to indicate critical areas with missing data (Figure 18).

Reference models of the bell tower of Vallecchia Church were built based on the respective point cloud, with the intention here of testing only completeness and, therefore, intending to avoid the influence of inaccuracies and possible deformations.

As presented in Section 4.2, despite the very high density of the Lixel L2Pro point cloud (over 21 million points), it provided a less accurate 3D model. Since its deformations exceed the threshold used to assess data completeness, comparison with the reference model constructed from X70 and VLX3 data yields insignificant results for completeness assessment. In this case, as we only wanted to consider the completeness, not the accuracy, of the model, the reference surfaces were adapted to the Lixel data. Moreover, to optimize processing time while preserving geometric detail, the Lixel point cloud was downsampled to approximately 10 million points, ensuring sufficient resolution for reliable comparison. The point clouds acquired with the X70GO and VLX 3 instruments did not require any downsampling, as their density and data distribution were already suitable for efficient processing.

The results obtained with X70GO, VLX 3, and Lixel L2 Pro are shown in Table 7. In all datasets, the percentage of missing data ranges from 13% in the VLX 3 dataset to 12% in both the X70GO and Lixel L2 Pro datasets. The missing data are primarily located in the upper part of the tower, where occlusions and scanning angles reduce the ability of the instruments to capture complete information. Despite this, the overall completeness of the datasets remains high.

**Figure 19:**
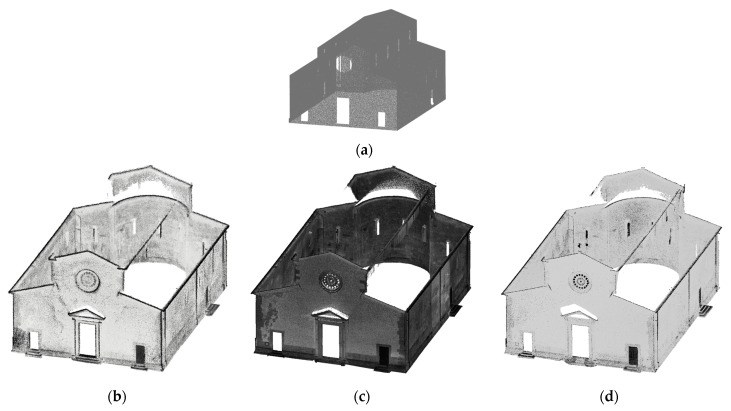
(**a**) Reference 3D model points cloud and dataset of (**b**) X70GO, (**c**) VLX 3 and (**d**) Lixel L2 Pro.

**Figure 20:**
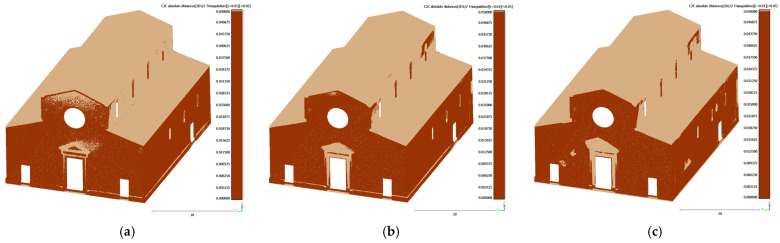
Density evaluation results: (**a**) X70GO, (**b**) VLX 3 and (**c**) Lixel L2 Pro.

For the Vallecchia Church, the completeness analysis was conducted exclusively on the exterior of the building; therefore, interior areas were removed from all datasets acquired with the three systems (Figure 19). The reference models of the Vallecchia Church were constructed based on their respective point clouds, for the same reasons outlined previously (Figure 20).

The results obtained with X70GO, VLX 3, and Lixel L2 Pro are shown in Table 8. As observed in the bell tower analysis, the missing data are predominantly located in the upper sections of the church and on the roof, where the limited scanning distance made it difficult to fully capture these areas. Among the three datasets, the VLX 3 dataset is the most complete, with 50% missing data, while the X70GO and Lixel L2 Pro datasets exhibit slightly higher proportions of missing data, at 55% and 54%, respectively.

**Figure 21:**
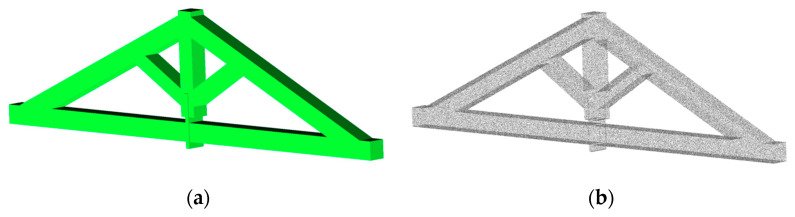
(**a**) Reference 3D model and (**b**) points cloud (299,988).

To specifically consider the effect of 3D model completeness on structural elements, a detailed C2C analysis was also performed on the truss of the Sorano Church. A 3D model was generated from the VLX 3 dataset, selected for its uniform density and good overall coverage (Figure 21).

The results obtained with X70GO and VLX 3 are shown in Table 9.

The C2C analysis proved to be an effective tool for detecting critical areas in the datasets and for verifying the geometric accuracy and completeness of the surveyed models. The results confirmed a high level of point cloud completeness, meeting the accuracy standards required for advanced modelling and structural simulation.

**Figure 22:**
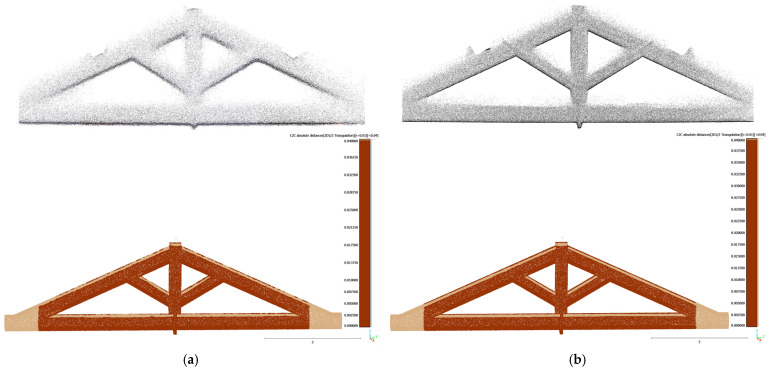
Testing dataset from (**a**) X70GO (183,775 points) and (**b**) VLX 3 (299,421 points).

Figure 18, Figure 20 and Figure 22, along with Table 7, Table 8 and Table 9 show that all three instruments yielded broadly comparable results in all three cases considered. The most significant discrepancies were observed in the upper portions of the tower, on the roof of the church, and in areas where the acquisition distance was too great or acquisition was not feasible. The variability of the paths followed during field acquisition, therefore, seems to have a limited influence on completeness in these cases, unlike the construction characteristics of the objects to be surveyed. Furthermore, the technological differences between the instruments considered are minimal in terms of the position of the “viewpoint” in relation to the surfaces to be surveyed, with the highest elements (and therefore those that are not in sight from the ground) remaining the most critical.

The completeness of the acquired point clouds, despite localized areas of missing data, is sufficient to derive 3D models suitable for numerical simulations. These models capture the essential geometry of the structural elements, which is all that is required for accurate numerical analysis, without the need to represent detailed architectural features. This demonstrates that even point clouds with partial data loss can provide a reliable basis for structural and geometrical assessments. Furthermore, features such as façade and lateral wall openings, the presence of buttresses, and vaults can all be identified from the point clouds and are necessary for the calculation of vulnerability indices.

## 5. Conclusions

Historic buildings require careful intervention to preserve both their formal and material aspects. Vulnerability risk assessment proceeds from preliminary analyses based primarily on direct observation and the collection of available documentary material, gradually moving towards more detailed numerical analyses that sometimes require invasive diagnostic investigations, such as soundings and corings, to correctly define the characteristics of the materials used in construction. Knowledge of the building is generally gained through direct in situ observation. This approach has certain limitations, including the need to reach the buildings, subjectivity in interpretation, and limitations in accessibility regarding viewpoint, lighting conditions, etc. This work proposes applying modern 3D surveying techniques at the early stages of investigation to overcome the limitations mentioned above. In fact, the 3D model can be considered as the digital representative of the real building and it can be shared offline or online to be observed simultaneously by several experts, allowing for a comparison of opinions and training activities for less experienced staff; the information provided by the intensity of the reflected signal allows details, textures, and materials to be recorded even in low light conditions; furthermore, the 3D data acquired can be used to obtain the metric information necessary for more detailed structural analysis and simulations.

Technological research oriented to the use of advanced techniques, increasingly efficient and at the same time easily accessible, is encouraging the development and experimentation of innovative operational tools for the documentation of cultural heritage. In recent years, rapid mapping has revolutionized surveying with the introduction of portable solutions, transforming the perspective of traditional 3D data acquisition methodologies.

Considering the objective of supporting structural analyses on the churches in the Lunigiana region, all the systems tested proved to speed up the fieldwork: this is an important element in this project, which intend to study over 27 churches in addition to the two cases presented in this paper and identified as prototypes.

All of the 3D models obtained describe the main dimensions of the buildings and their elements (e.g., openings, lintels, trusses, etc.) that must be analysed for the assessment of seismic risk vulnerability; small details, often relevant for structural analysis (as it is for crack patterns), are, in general, not recognisable on the 3D model. Nevertheless, high-resolution images profitably supply detailed surface interpretation. A common trend is to enhance images not only to obtain texturized 3D models but also to offer interactive model visualisation and exploration systems based on 360° panoramas or gaussian splatting technology, highlighting a notable complementarity between the metric and communicative value of the 3D models produced.

While this study does not directly address the documentation of movable heritage within the churches, it recognizes that SLAM systems could have the potential to support the spatial localization and preservation of such movable assets, contributing to a more comprehensive protection strategy.

Hardware systems require the use of proprietary software, which is characterised by a simple interface but offers limited control over the process and lacks technical documentation. Almost no settings are available to personalise the scan process in the field. The trajectory planning is based on common sense, on the need to ensure an overlap zone between the start and finish points (or to station exactly on the same marker as the start and end point), to repeat the scanning of relevant elements coming from multiple directions, and to verify already during the survey that adequate coverage has been obtained.

Residual distances referred to targets distributed across the sites are considered to be effective for evaluating different trajectory constraint methods and model georeferencing (Section 4.1). Planimetric residuals are worse than vertical, which are almost negligible. Considering the absolute distances in XY, the higher residuals at the 95th percentile are slightly less than 4 cm (median values vary from 6 mm to 17 mm). It is worth mentioning that in some projects the same statistic is about 1.5 cm.

To globally evaluate the model’s accuracy, all the point clouds resulting from different sensors and georeferencing strategies are compared to a ground truth obtained through a total station survey (Section 4.2). Also in this case, the planimetric residuals are more relevant than verticals, which are on the order of a centimetre. Moreover, georeferencing via GCPs guarantees greater accuracy than using GNSS alone; an interesting approach could be to integrate both information into the processing phase, whereas currently the software tested only allows for their alternative use.

This test highlights, from a metric point of view, the different performances of the instruments considered: the best results are those obtained with the VLX 3, which, particularly in the Sorano Church, show residual distances similar to those expected from a TLS system.

The X70GO was tested more thoroughly and in a wider variety of operating conditions; the median residual distance reached 2 cm in only one case, with a corresponding RMSE of approximately 3 cm.

The dataset acquired by the Lixel L2 Pro presents the highest residual, and the 3D model appears slightly deformed.

Although this does not alter the metric characteristics of the 3D model, point oversampling is generally applied during the processing phase, thereby making the initially detected points denser and allowing for better visualisation, particularly of the textured model. It is challenging to recognise how the final model is affected by raw data (i.e., effects related to sensors) or by post-processing (i.e., effects related to software). For this reason, assessments have been carried out considering self-reference aspects, as point cloud density, and also subjective 3D model interpretations. Mains architectural elements relevant for structural studies can be recognised (and measured) in all the dataset, but their interpretation and detailed analysis was not always possible. More minute details, which are nevertheless of interest for structural purposes, such as cracks and fractures, are only recognisable in some cases. In general, it is beneficial to be able to assess deformations, misalignments, and out-of-plumb of elements such as walls, columns, lintels, etc.

Among the tested systems, VLX 3 demonstrates the highest accuracy and consistency in data quality, also considering longer distances, outperforming both Lixel L2 Pro and X70GO. The data acquired by the Lixel L2 Pro instrument demonstrate a lower overall accuracy compared to the other instruments used. Despite this, the data visualisation is effective and point clouds are still acceptable for applications where high metric precision is not required.

## Figures and Tables

**Table 1:** Main specs of the devices tested in the study.

	X70GO	VLX 3	Lixel L2 Pro
Device configuration	Handheld	Wearable	Handheld
N° LiDAR	1	2	1
Points per second	200,000 pts/s	2 × 1,280,000 pts/s	640,000 pts/s
N° of cameras	2	4	2
Visual SLAM	yes	No	yes
Camera resolution	12 MP	20 MP	48 MP
RTK GNSS	Integrable	No	Integrated
Declared accuracy	6 mm	5 mm	5 mm
Processing software	GOpost	NavVis IVION (cloud-based)	LixelStudio

**Table 2:** Summary of key software features.

	GOpost	NavVis IVION	LixelStudio
Manufacturer	Stonex	NavVis	XGRIDS
Post-elaboration	Desktop (Windows)	Cloud	Desktop (Windows)
Georeferencing	RTK–GCP	GCP	RTK–GCP
Colouring	yes	yes	yes
Densification	Yes, up to version 66; optional for newer versions	Down- or over-sampling option	Optional
Filtering	Persons removal	Blurring of faces, bodies and plates	Moving objects removal

**Table 3:** Time for the acquisition and the post-processing (it also includes the initialization time, if present).

	X70GO	VLX 3	Lixel L2 Pro
Paths	6	2	1
Acquisition time	6–12 min	18–25 min	20 min
Processing time	1–2 h	4 h	2 h

**Table 4:** Summary of the acquisition paths considered in the test and the georeferencing strategies adopted.

	Instrument	Path	Georeferencing	# Target	Trajectory
Sorano Church	VLX 3	S01/25	Targets	10	500 m
	X70GO	S03/24	Targets	9	140 m
				4	
		S04/24	Targets	6	140 m
		S05/24	Targets	3	70 m
		S01/25	Targets	10	180 m
				5	
			RTK GNSS	-	180 m
		S02/24	RTK GNSS	-	300 m
		S06/24	RTK GNSS	-	150 m
Vallecchia Church	VLX 3	V01/25	Targets	9	450 m
	X70GO	V01/25	Targets	10	340 m
				4	
			RTK GNSS	-	
	Lixel L2 Pro	V01/25	Targets	9	450 m

**Table 5:** Residual distances on targets.

	Path	Target		Planimetric Residuals (XY, Absolute Values) [m]					Vertical Residuals (Z, Signed Values) [m]		
				Mean	Median	RMSE	68th %tile	95th %tile	Mean	σ	RMSE
Sorano Church	VLX 3 S01/25	GCPs	10	0.003	0.001	0.004	0.002	0.013	0.000	0.001	0.008
		CPs	-	-	-	-	-	-	-	-	-
	X70GO S03/24	GCPs	9	0.014	0.013	0.015	0.015	0.025	0.000	0.008	0.008
		CPs	-	-	-	-	-	-	-	-	-
		GCPs	4	0.008	0.008	0.009	0.010	0.013	0.000	0.002	0.002
		CPs	5	0.015	0.013	0.012	0.013	0.015	−0.004	0.006	0.007
	X70GO S04/24	GCPs	6	0.006	0.006	0.006	0.007	0.008	0.000	0.002	0.002
		CPs	-	-	-	-	-	-	-	-	-
	X70GO S05/24	GCPs	3	0.007	0.006	0.008	0.008	0.010	0.000	0.000	0.000
		CPs	-	-	-	-	-	-	-	-	-
	X70GO S01/25	GCPs	10	0.013	0.011	0.017	0.014	0.032	0.000	0.006	0.006
		CPs	-	-	-	-	-	-	-	-	-
		GCPs	5	0.024	0.017	0.026	0.035	0.038	0.000	0.004	0.004
		CPs	5	0.017	0.014	0.020	0.017	0.036	−0.003	0.008	0.009
Vallecchia Church	VLX 3	GCPs	9	0.001	0.001	0.004	0.001	0.002	0.000	0.001	0.001
		CPs	-	-	-	-	-	-	-	-	-
	X70GO V01/25	GCPs	10	0.011	0.010	0.013	0.010	0.031	0.000	0.010	0.010
		CPs	-	-	-	-	-	-	-	-	-
		GCPs	4	0.006	0.006	0.007	0.007	0.008	0.000	0.000	0.000
		CPs	6	0.013	0.011	0.016	0.014	0.031	−0.003	0.015	0.016

**Table 6:** Comparison of SLAM-based 3D models with respect to a total station measured sparse point cloud: the table summarizes the results of 3D models processed following different strategies: supported by GNSS or by target (only with GCPs and with CPs).

	Path	Target (GCPs/CPs) or GNSS	Planimetric Residuals (XY, Absolute Values) [m]					Vertical residuals (Z, Signed Values) [m]		
			Mean	Median	RMSE	68th %tile	95th %tile	Mean	σ	RMSE
Sorano Church	X70GO S02/24	GNSS	0.004	0.014	0.006	0.020	0.039	0.000	0.005	0.005
	X70GO S03/24	9/0	0.019	0.010	0.025	0.017	0.040	0.008	0.021	0.023
		4/5	0.020	0.012	0.029	0.019	0.051	0.009	0.022	0.024
	X70GO S04/24	6/0	0.017	0.014	0.024	0.020	0.039	0.008	0.022	0.024
	X70GO S05/24	3/0	0.015	0.007	0.021	0.014	0.041	0.007	0.018	0.020
	X70GO S06/24	GNSS	0.017	0.017	0.019	0.021	0.030	0.000	0.010	0.010
	X70GO S01/25	10/0	0.020	0.012	0.026	0.020	0.047	0.008	0.020	0.022
		5/5	0.018	0.010	0.024	0.017	0.046	0.009	0.022	0.024
		GNSS	0.025	0.021	0.033	0.037	0.058	−0.003	0.020	0.014
	VLX 3 2025	10/0	0.015	0.007	0.024	0.019	0.052	0.009	0.022	0.025
Vallecchia church	X70GO V01/25	10/0	0.011	0.005	0.018	0.009	0.030	0.000	0.005	0.005
		4/6	0.007	0.005	0.009	0.010	0.018	0.000	0.001	0.001
		GNSS	0.016	0.013	0.020	0.018	0.036	−0.003	0.015	0.015
	VLX 3 2025	9/0	0.005	0.004	0.006	0.006	0.015	0.000	0.001	0.001
	Lixel L2 Pro 2025	9/0	0.074	0.080	0.075	0.085	0.088	0.003	0.010	0.011

**Table 7:** Vallecchia Church—Belltower: results of the analysis performed; points beyond the set threshold (5 cm) are considered to correspond to ‘missing’ data in the point model.

	Dataset	Missing Dataset
X70GO	4,487,135	926,905 (12%)
VLX 3	6,951,798	1,048,059 (13%)
Lixel L2 Pro	10,027,081	982,476 (12%)

**Table 8:** Vallecchia Church—Church: results of the analysis performed.

	Dataset	Missing Dataset
X70GO	12,932,352	4,370,866 (55%)
VLX 3	22,099,022	4,011,038 (50%)
Lixel L2 Pro	19,294,359	4,332,166 (54%)

**Table 9:** Sorano Church, truss: results of C2C analysis.

	Dataset	Missing Dataset
X70GO	145,903	75,370 (25%)
VLX 3	225,472	73,187 (24%)

## Data Availability

The raw data supporting the conclusions of this article will be made available by the authors on request.

## References

[1] Calantropio A., Chiabrando F., Sammartano G., Spanò A., Teppati Losè L. (2018). UAV strategies validation and remote sensing data for damage assessment in post-disaster scenarios. ISPRS-Int. Arch. Photogramm. Remote Sens. Spat. Inf. Sci..

[2] Girelli V.A. (2007). Tecniche Digitali per il Rilievo, la Modellazione Tridimensionale e la Rappresentazione Nel Campo dei Beni Culturali. Ph.D. Thesis.

[3] Malinverni E.S., Pierdicca R., Bozzi C.A., Bartolucci D. Evaluating a slam-based mobile mapping system: A methodological comparison for 3D heritage scene real-time reconstruction. Proceedings of the 2018 Metrology for Archaeology and Cultural Heritage (MetroArchaeo).

[4] Zhang Y., Carballo A., Yang H., Takeda K. (2023). Perception and sensing for autonomous vehicles under adverse weather conditions: A survey. ISPRS J. Photogramm. Remote Sens..

[5] How NeRFs and 3D Gaussian Splatting are Reshaping SLAM: A Survey. (n.d.). https://fabiotosi92.github.io/files/survey-slam.pdf.

[6] Sammartano G., Spanò A. (2018). Point clouds by SLAM-based mobile mapping systems: Accuracy and geometric content validation in multisensor survey and stand-alone acquisition. Appl. Geomat..

[7] Ioannides M., Fink E., Anderson J., Fresa A., Antony Cassar A., Münster S. (2026). 3D research challenges in cultural heritage VI: Digital twin versus memory twin. Lecture Notes in Computer Science.

[8] Fan Z., Zhang L., Wang X., Shen Y., Deng F. (2025). LiDAR, IMU, and camera fusion for simultaneous localization and mapping: A systematic review. Artif. Intell. Rev..

[9] Debeunne C., Vivet D. (2020). A review of visual-LiDAR Fusion based simultaneous localization and mapping. Sensors.

[10] Yarovoi A., Cho Y.K. (2024). Review of simultaneous localization and mapping (SLAM) for construction robotics applications. Autom. Constr..

[11] Chen P., Zhao X., Zeng L., Liu L., Liu S., Sun L., Li Z., Chen H., Liu G., Qiao Z. (2025). A Review of Research on SLAM Technology Based on the Fusion of LiDAR and Vision. Sensors.

[12] Toschi I., Rodríguez-Gonzálvez P., Remondino F., Minto S., Orlandini S., Fuller A. (2015). accuracy evaluation of a mobile mapping system with advanced statistical methods. Int. Arch. Photogramm. Remote Sens. Spat. Inf. Sci..

[13] Bronzino G.P.C., Grasso N., Matrone F., Osello A., Piras M. (2019). Laser-visual-inertial odometry based solution for 3D heritage modeling: The Sanctuary of the Blessed Virgin of Trompone. Int. Arch. Photogramm. Remote Sens. Spat. Inf. Sci..

[14] Tucci G., Visintini D., Bonora V., Parisi E.I. (2018). Examination of indoor mobile mapping systems in a diversified internal/external test field. Appl. Sci..

[15] Rabbia A., Sammartano G., Spanò A. Fostering Etruscan heritage with effective integration of UAV, TLS and SLAM-based methods. Proceedings of the 2020 IMEKO TC-4 International Conference on Metrology for Archaeology and Cultural Heritage.

[16] La Guardia M., Masiero A., Bonora V., Alessandrini A. (2025). Open source deep learning solutions for the classification of MMS urban 3D data. Int. Arch. Photogramm. Remote Sens. Spat. Inf. Sci..

[17] Tanduo B., Chiabrando F., Coluccia L., Auriemma R. (2023). Underground heritage documentation: The case study of Grotta Zinzulusa in Castro (Lecce-Italy). Int. Arch. Photogramm. Remote Sens. Spat. Inf. Sci..

[18] Zlot R., Bosse M., Greenop K., Jarzab Z., Juckes E., Roberts J. (2014). Efficiently capturing large, complex cultural heritage sites with a handheld mobile 3D laser mapping system. J. Cult. Herit..

[19] Chiabrando F., Della Coletta C., Sammartano G., Spanò A., Spreafico A. (2018). “Torino 1911” project: A contribution of a slam-based survey to extensive 3D heritage modeling. Int. Arch. Photogramm. Remote Sens. Spat. Inf. Sci..

[20] Gharineiat Z., Tarsha Kurdi F., Henny K., Gray H., Jamieson A., Reeves N. (2024). Assessment of NavVis VLX and BLK2GO SLAM scanner accuracy for outdoor and indoor surveying tasks. Remote Sens..

[21] Barba S., Ferreyra C., Cotella V.A., di Filippo A., Amalfitano S., Rauterberg M. (2021). A SLAM integrated approach for digital heritage documentation. Culture and Computing. Interactive Cultural Heritage and Arts.

[22] Lehtola V.V., Kaartinen H., Nüchter A., Kaijaluoto R., Kukko A., Litkey P., Hyyppä J. (2017). Comparison of the selected state-of-the-art 3D indoor scanning and point cloud generation methods. Remote Sens..

[23] Buill F., Núñez-Andrés M.A., Costa-Jover A., Moreno D., Puche J.M., Macias J.M. (2020). Terrestrial laser scanner for the formal assessment of a Roman-medieval structure—the cloister of the cathedral of Tarragona (Spain). Geosciences.

[24] Costantino D., Rossi G., Pepe M., Leserri M. (2023). Experiences of TLS, terrestrial and UAV photogrammetry in Cultural Heritage environment for restoration and maintenance purposes of Royal Racconigi castle, Italy. Proceedings of the 2022 IMEKO TC4 International Conference on Metrology for Archaeology and Cultural Heritage.

[25] Tucci G., Bonora V. (2017). Towers in San Gimignano: Metric survey approach. J. Perform. Constr. Facil..

[26] Hamzić A., Kulo N., Đidelija M., Topoljak J., Mulahusić A., Tuno N., Ademović N. (2025). Assessment of Minaret Inclination and Structural Capacity Using Terrestrial Laser Scanning and 3D Numerical Modeling: A Case Study of the Bjelave Mosque. Geomatics.

[27] Mulahusić A., Tuno N., Topoljak J., Gačanović F., Karabegović I., Kovačević A., Mandžuka S. (2022). Integration of UAV and terrestrial photogrammetry for cultural and historical heritage recording and 3D modelling: A case study of the ‘Sebilj’ Fountain in Sarajevo, Bosnia and Herzegovina. New Technologies, Development and Application V. NT 2022.

[28] Furini A., Bolognesi M. (2014). Accuracy of cultural heritage 3D models by RPAS and terrestrial photogrammetry. ISPRS Int. Arch. Photogramm. Remote Sens. Spat. Inf. Sci..

[29] Alshawabkeh Y., Baik A., Miky Y. (2021). Integration of laser scanner and photogrammetry for heritage BIM enhancement. ISPRS Int. J. Geo-Inf..

[30] Pérez-García J.L., Gómez-López J.M., Mozas-Calvache A.T., Delgado- García J. (2024). Analysis of the photogrammetric use of 360-degree cameras in complex heritage-related scenes: Case of the Necropolis of Qubbet el-Hawa (Aswan Egypt). Sensors.

[31] Đurić I., Obradović R., Vasiljević I., Ralević N., Stojaković V. (2021). Twodimensional shape analysis of complex geometry based on photogrammetric models of iconostases. Appl. Sci..

[32] Lerma J.L., Navarro S., Cabrelles M., Villaverde V. (2010). Terrestrial laser scanning and close range photogrammetry for 3D archaeological documentation: The Upper Palaeolithic Cave of Parpalló as a case study. J. Archaeol. Sci..

[33] Di Stefano F., Chiappini S., Gorreja A., Balestra M., Pierdicca R. (2021). Mobile 3D scan LiDAR: A literature review. Geomat. Nat. Hazards Risk.

[34] Fillia E., Sammartano G., Tocci C. Spanò Modellare la conoscenza della vulnerabilità sismica delle chiese in muratura storica con tecnologie 3D speditive. Proceedings of the Conferenza ASITA 2021.

[35] Sammartano G., Avena M., Fillia E., Spanò A. (2023). Integrated HBIM-GIS Models for Multi-Scale Seismic Vulnerability Assessment of Historical Buildings. Remote Sens..

[36] Todisco P., Ciancio V., Nastri E. (2024). Seismic performance assessment of the church of SS. Annunziata in Paestum through finite element analysis. Eng. Fail. Anal..

[37] Tanduo B., Teppati Losè L., Chiabrando F. (2023). Documentation of complex environments in cultural heritage sites. A slam-based survey in the Castello del Valentino basement. Int. Arch. Photogramm. Remote Sens. Spat. Inf. Sci..

[38] Yiğit A.Y., Gamze Hamal S.N., Ulvi A., Yakar M. (2023). Comparative analysis of mobile laser scanning and terrestrial laser scanning for the indoor mapping. Build. Res. Inf..

[39] Pepe M., Alfio V.S., Costantino D., Herban S. (2022). Rapid and accurate production of 3D point cloud via latest-generation sensors in the field of cultural heritage: A Comparison between SLAM and Spherical Videogrammetry. Heritage.

[40] Betti M., Bonora V., Galano L., Pellis E., Tucci G., Vignoli A. (2021). An Integrated geometric and material survey for the conservation of heritage masonry structures. Heritage.

[41] He X., Chen Y., Zhou Z., Ke K. (2024). Fuse replacement implementation by shaking table tests on hybrid moment-resisting frame. J. Build. Eng..

[42] Kujawa M., Lubowiecka I., Szymczak C. (2020). Finite element modelling of a historic church structure in the context of a masonry damage analysis. Eng. Fail. Anal..

[43] (2011). DPCM 2011 Direttiva del Presidente del Consiglio dei Ministri per la Valutazione e Riduzione del Rischio Sismico del Patrimonio Culturale con Riferimento alle NTC 2008 (Directive of the President of the Council of Ministers for the Evaluation and Reduction of Seismic Risk of Cultural Heritage with Reference to the 2008 NTCs). G. U. n. 47 del 26.02.2011. https://www.gazzettaufficiale.it/eli/id/2011/02/26/11A02374/sg.

[44] Lourenço P.B., Oliveira D.V., Leite J.C., Ingham J.M., Modena C., Da Porto F. (2013). Simplified indexes for the seismic assessment of masonry buildings: International database and validation. Eng. Fail. Anal..

[45] Colapietro M. (2022). Analisi Della Risposta e Sicurezza Sismica del Campanile di San Lorenzo a Minucciano (Analysis of the Response and Seismic Safety of the Bell Tower of San Lorenzo in Minucciano). Barchelor’s Thesis in Architecture.

[46] BeWeb (Beni Ecclesiastici in Web—Beni Ecclesiastici in Web) Online Database of the Italian Catholic Church. https://beweb.chiesacattolica.it/?l=it_IT.

[47] Cadena C., Carlone L., Carrillo H., Latif Y., Scaramuzza D., Neira J., Reid I., Leonard J.J. (2016). Past, present, and future of simultaneous localization and mapping: Toward the robust-perception age. IEEE Trans. Robot..

[48] Conti A., Pagliaricci G., Bonora V., Tucci G. (2024). A comparison between terrestrial laser scanning and hand-held mobile mapping for the documentation of built heritage. Int. Arch. Photogramm. Remote Sens. Spat. Inf. Sci..

[49] CloudCompare, 3D Point Cloud and Mesh Processing Software, Open Source Project. Homepage. https://cloudcompare.org/index.html.

[50] Diaz V., Van Oosterom P., Meijers M., Verbree E., Ahmed N., Van Lankveld T., Kolbe T.H., Donaubauer A., Beil C. (2024). Comparison of Cloud-to-Cloud Distance Calculation Methods—Is the Most Complex Always the Most Suitable?. Recent Advances in 3D Geoinformation Science.

[51] Lague D., Brodu N., Leroux J. (2013). Accurate 3D comparison of complex topography with terrestrial laser scanner: Application to the Rangitikei canyon (N-Z). ISPRS J. Photogramm. Remote Sens..

[52] Yiğit A.Y., Ulvi A. (2022). Comparison of the Wearable Mobile Laser Scanner (WMLS) with Other Point Cloud Data Collection Methods in Cultural Heritage: A Case Study of Diokaisareia. ACM J. Comput. Cult. Herit..

[53] Bonfanti C., Patrucco G., Perri S., Sammartano G., Spanò A. (2021). A new indoor lidar-based mms challenging complex architectural environments. Int. Arch. Photogramm. Remote Sens. Spat. Inf. Sci..

[54] Liao Z., Dong X., He Q. (2024). Calculating the optimal point cloud density for airborne lidar landslide investigation: An adaptive approach. Remote Sens..

[55] Boardman C., Bryan P., McDougall L., Reuter T., Payne E., Moitinho V., Rodgers T., Honkova J., O’Connor L., Blockley C. (2018). 3D Laser Scanning for Heritage: Advice and Guidance on the Use of Laser Scanning in Archaeology and Architecture.

[56] Sammartano G., Patrucco G., Avena M., Bonfanti C., Spanò A. (2024). Enhancing terrestrial point clouds using upsampling strategy: First observation and test on faro flash technology. Int. Arch. Photogramm. Remote Sens. Spat. Inf. Sci..

[57] Li L., Zhang X. (2025). A robust assessment method of point cloud quality for enhancing 3D robotic scanning. Robot. Comput. Integr. Manuf..

[58] Zhang S., Liu C., Haala N. (2024). Guided by model quality: UAV path planning for complete and precise 3D reconstruction of complex buildings. Int. J. Appl. Earth Obs. Geoinf..

[59] Chen S., Fan G., Li J. (2023). Improving completeness and accuracy of 3D point clouds by using deep learning for applications of digital twins to civil structures. Adv. Eng. Inform..

[60] Schnabel R., Degener P., Klein R. (2009). Completion and reconstruction with primitive shapes. Comput. Graph. Forum.

[61] Achlioptas P., Diamanti O., Mitliagkas I., Guibas L. (2018). Learning representations and generative models for 3d point clouds. Proceedings of the 35th International Conference on Machine Learning.

[62] Sipiran I., Mendoza A., Apaza A., Lopez C. (2022). Data-driven restoration of digital archaeological pottery with point cloud analysis. Int. J. Comput. Vis..

[63] Ioannides M., Patias P., Ioannides M., Patias P. (2023). The complexity and quality in 3D digitisation of the past: Challenges and risks. 3D Research Challenges in Cultural Heritage III.

[64] Spurek P., Kasymov A., Mazur M., Janik D., Tadeja S.K., Tabor J., Trzciński T. Hyper Pocket: Generative point cloud completion. Proceedings of the 2022 IEEE/RSJ International Conference on Intelligent Robots and Systems (IROS).

